# Improved Dielectric Breakdown Strength of Polyimide by Incorporating Polydopamine-Coated Graphitic Carbon Nitride

**DOI:** 10.3390/polym14030385

**Published:** 2022-01-19

**Authors:** Yinjie Dong, Zhaoyang Wang, Shouchao Huo, Jun Lin, Shaojian He

**Affiliations:** State Key Laboratory of Alternate Electrical Power System with Renewable Energy Sources, North China Electric Power University, Beijing 102206, China; ddxdyj@126.com (Y.D.); wang94269264@163.com (Z.W.); huo472@foxmail.com (S.H.); jun.lin@ncepu.edu.cn (J.L.)

**Keywords:** polyimide, graphitic carbon nitride nanosheets, polydopamine, interfacial interaction, breakdown strength

## Abstract

Breakdown strength is an important parameter for polymer dielectric, and introducing inorganic filler into the polymer matrix is an efficient method to improve the breakdown strength. In this work, graphitic carbon nitride nanosheets (CNNS) were ultrasonically exfoliated and coated with polydopamine to obtain modified nanosheets (DCNNS), and then polyimide (PI) composite films with various CNNS and DCNNS were prepared and compared. Owing to the abundant hydroxyl groups of polydopamine, good filler-polymer compatibility and uniform filler dispersion were achieved for PI/DCNNS composites. Both breakdown strength and dielectric constant were improved with the addition of either CNNS or DCNNS. However, at the same filler content, the PI/DCNNS composites exhibited higher breakdown strength and dielectric constant than the PI/CNNS. The PI composite with 0.5 wt% DCNNS showed the highest breakdown strength of ~300 kV/mm, increased by 67.6% as compared to the pure PI, while the PI/CNNS composite with the same filler content only increased by 14.5%.

## 1. Introduction

Polymer dielectrics with good dielectric properties and high-energy storage density play an important role in modern information and electronic industries connected to, for example, charge storage devices and embedded capacitors [1,2]. However, with the rapid development of the modern power industry, more requirements are proposed on the stability of polymer dielectrics and electronic devices operating under high electrical conditions. For the insulation dielectric materials, once the surface flashover or body breakdown phenomenon occurs, it can lead to insulation dielectric surface degradation and an equipment short circuit, ultimately threatening the operational reliability of the power equipment. What is more, according to the formula of energy storage density of the linear polymer, $U=\frac{1}{2}\varepsilon_{0}\varepsilon_{r}E_{b}^{2}$, the energy storage density (*U*) is proportional to the square of breakdown strength (*E_b_*) tolerated by the film, so it is of great significance to improve the breakdown strength of polymer in the power industry [3,4].

Polyimide (PI), as a typical engineering polymer material, has been widely used in insulation materials, microelectronics, aerospace and other fields in recent years due to its high thermal stability, excellent electrical insulation, and mechanical properties [5,6,7]. However, the defects (such as impurities and micro-pores) during the breakdown of pure PI will lead to a theoretical reduction in the actual breakdown strength of the material, which limits its demand in some special applications [8]. The traditional strategy of adding inorganic nanofillers (such as Al_2_O_3_ [9,10] and BaTiO_3_ [11]) to the polymer matrix can improve the comprehensive properties of the composites and suppress the distorted electric field. However, the small particle size and large specific surface area of conventional nanoparticles lead to their poor dispersion in polymers. Recently, it was reported that two-dimensional nanofillers [12,13,14,15,16,17,18,19], such as boron nitride, graphene, mica and titanium dioxide nanosheets, play an important role in improving the performance, including the breakdown field strength, of polymer composites when they are evenly dispersed in the matrix.

Two-dimensional graphitic carbon nitride (g-C_3_N_4_) is a promising material commonly used in the fields of photocatalysis and heterogeneous catalysis due to its simple synthesis and low cost [20,21,22]. Recently, Zhu et al. [23] reported that the frictional properties of the composites were improved by introducing g-C_3_N_4_ as a filler in the polyimide matrix. This work provides an example of the application of g-C_3_N_4_ as an excellent nanofiller in polymer-based composites. Wang et al. [24] explored the potential application of carbon nitride nanosheets in improving the thermal conductivity of PI through experiments and simulations. The improvement in thermal conductivity benefited from the self-orientation and strong interaction of the fillers, along with the PI film. Although g-C_3_N_4_ has been used as a filler to improve polymers’ properties, the research on its application as an electrical insulation filler in the field of high-voltage insulation is relatively rare [25]. Generally, the poor interfacial compatibility between the polymer matrix and the filler affects the dielectric breakdown strength of composites [26,27,28]. To solve this problem, an economical, efficient and easy way is to modify the inorganic fillers through surface treatment, modulating the interfacial properties and increasing the breakdown field strength of the composite dielectric. In our previous work [25], silicone rubber (SR)/g-C_3_N_4_ composites were prepared by in situ modification with vinyl tri-methoxysilane (VTMS), and the incorporation of VTMS reduced the defects in the prepared composites and improved their breakdown strength and mechanical properties.

The polydopamine coating is formed by spontaneous oxidative copolymerization of the dopamine on the surface of the substrate. Compared with traditional chemical modification methods, polydopamine can adhere to the surface of most materials without destroying the matrix structure. In addition, strong adhesion is formed between the polydopamine-encapsulated nanofiller and the polymer [13]. The covalent bonds are formed between the dicarboxylic anhydride of PI and the amine groups of polydopamine, further improving the compatibility of carbon nitride and the PI matrix.

In this work, the g-C_3_N_4_ composites were prepared from melamine through thermal condensation, and then carbon nitride nanosheets (CNNS) were prepared using the ultrasonic stripping method. Carbon nitride nanosheets modified by polydopamine (DCNNS) were prepared by self-polymerization of dopamine in a weak alkali environment, and then PI composite films with various CNNS and DCNNS were prepared by solution casting. The microstructure, dielectric, and breakdown properties of the composite films were investigated.

## 2. Materials and Methods

### 2.1. Materials

Melamine was purchased from Anhui Jinhe Co., Ltd. (Chuzhou, China). 4,4-oxydianiline (ODA) was purchased from Tianjin Guangfu Fine Chemical Research Institute (Tianjin, China). Pyromellitic dianhydride (PMDA) was purchased from Beijing Chemical Factory (Beijing, China). Dimethylacetamide (DMAc) was purchased from Beijing Innochem Science & Technology Co., Ltd. (Beijing, China). Tris(hydroxymethyl)aminomethane hydrochloride (Tris-HCl) and dopamine hydrochloride were purchased from Alfa Aesar Co., Ltd. (Shanghai, China). All reagents were used as received.

### 2.2. Sample Preparation

Preparation of the CNNS: Melamine powder covered with thin aluminum foil was heated to 500 °C with a heating rate of 3 °C·min^−1^ in a muffle furnace, and the temperature was maintained at 550 °C for 4 h. After cooling to room temperature, the bulk g-C_3_N_4_ was obtained. The ground bulk g-C_3_N_4_ (10 g) was added into 1 L of deionized water and then stirred with a high-speed mixer at a speed of 13,000 rpm for 2 h, followed by ultrasonically treating for 48 h. After standing for 5 h, the exfoliated CNNS was left in the upper white suspension. After evaporating most of the water at 80 °C, the upper suspension was concentrated from 1 L to 50 mL, and then the CNNS powder was obtained through freeze-drying.

Preparation of the DCNNS: The CNNS powder (4 g) was ultrasonically dispersed in 100 mL of deionized water for 30 min, and then 0.315 g of Tri-HCl and 0.379 g of dopamine hydrochloride was added to the CNNS aqueous suspension. After adjusting the pH to 8.5 by NaOH, the suspension was stirred for 4 h to complete the modification. Finally, the mixture was centrifuged and rinsed repeatedly 5 times followed by freeze-drying to obtain the DCNNS powder.

Preparation of the PI composite films: The PI/CNNS and PI/DCNNS composite films were prepared through a viscous prepolymer cast on the glass slide and the thermal imidization method. A certain amount of CNNS or DCNNS was ultrasonically dispersed in 30 mL of DMAC for 2 h, and then equimolar proportions of 3.064 g of ODA and 3.34 g of PMDA were added and stirred until the ODA was dissolved completely. Subsequently, the mixture was stirred under a nitrogen flow for 8 h, followed by degassing under vacuum for 2 h. The solid content of the PAA/CNNS and PAA/DCNNS suspension was 18%. After casting on a flat glass plate, the mixture was converted into films by scraping them with a glass rod, and then thermally imidized at 60 °C for 10 h, 120 °C, 200 °C, 250 °C and 320 °C for 1 h each. After the sample was naturally cooled to room temperature, the yellow and transparent PI composite films were obtained and denoted as PI/CNNS-X and PI/DCNNS-Y, where X% and Y% stand for the weight percentage of the CNNS and DCNNS. The thickness of the PI composites was approximately 30 μm.

### 2.3. Characterization and Measurement

The fractured surface of the PI/CNNS and PI/DCNNS composite films were observed by scanning electron microscopy (SEM, SU8010, Hitachi, Tokyo, Japan) with an accelerating voltage of 10 kV. The surface element composition was determined by the X-ray photoelectron spectrometer (XPS, Thermo Scientific K-Alpha, Waltham, MA, USA) equipped with an Al Ka X-ray source under an operating pressure below 8 × 10^−10^ Pa. The dielectric properties were tested by precision dielectric test apparatus (Novocontrol Concept 80, Montabaur, Germany). The dielectric breakdown strength (*E_b_*) was tested using a voltage-withstand testing device (HCDJC; Beijing Huace Testing Instrument Co. Ltd., Beijing, China) at ambient temperature, with an increasing alternating voltage of 0.5 kV/s. The specimens were sandwiched between two copper rod electrodes with diameters of 25 mm and immersed in pure silicone oil to prevent surface flashover.

## 3. Results and Discussions

### 3.1. Structure of the CNNS and DCNNS

**Figure 1:**
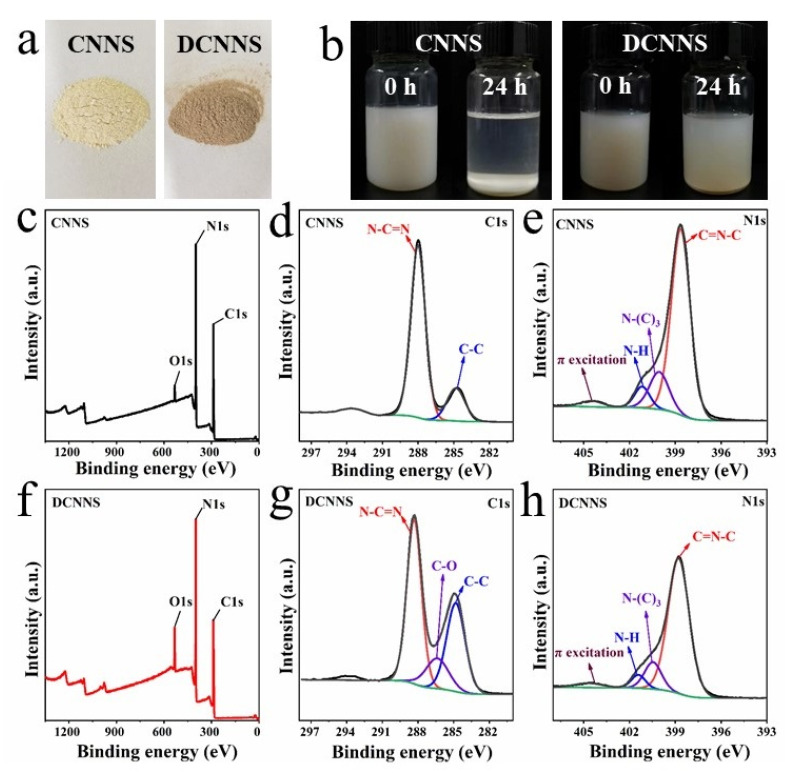
The photographs of (**a**) CNNS and DCNNS powder; (**b**) dispersing stability of CNNS and DCNNS in DMAC; XPS (**c**) wide-scan, (**d**) C1s and (**e**) N1s core-level spectra of CNNS; XPS (**f**) wide-scan, (**g**) C1s and (**h**) N1s core-level spectra of DCNNS.

The photographs of the CNNS and DCNNS are shown in Figure 1a. It can be seen that the color of the CNNS powder is dark-yellow, while it changes to gray-brown when coated with polydopamine. As shown in Figure 1b, the DCNNS can form a very stable suspension in the DMAc, and no noticeable precipitate was observed even after 24 h. In contrast, the CNNS were very unstable in the DMAc, as all the CNNS settled to the bottom after 24 h. Since polydopamine with many hydroxyl groups is coated on the surfaces of the nanosheets, the DCNNS can form hydrogen bonds with the DMAc, which effectively improves the dispersion and stability of the nanosheets in the solvent.

XPS measurement was used to investigate the surface chemical composition of the elements in the CNNS and DCNNS. As compared between Figure 1c,f, both the CNNS and DCNNS contained the elements C, O, and N, while the DCNNS had more O element as compared to the CNNS. Owing to the numerous oxygen-containing functional groups, the content of the O element increased from 3.28% to 7.44%, and the C/O ratio decreased from 12.4 to 6.3 after the polydopamine was coated on the nanosheets. In Figure 1d, the C1s spectra of both the CNNS and DCNNS contained two peaks at 284.8 eV and 288.0 eV, which were assigned to C-C and N-C=N, respectively. The dominant peak of N-C=N originated from the aromatic nitrogen heterocyclic structure of the g-C_3_N_4_ [29]. However, one more peak at 286.1 eV related to the C-O peak was found in the spectrum of the DCNNS, which should be attributed to the amidogen and phenolic hydroxyl from the polydopamine. In Figure 1e, the N1s spectrum of the CNNS presented four component peaks, C=N-C at 398.6 eV, N-(C)_3_ at 400.1 eV, N-H at 401.2 eV, and a heterocyclic charging effect of 404.5 eV [30]. In contrast to the CNNS, the DCNNS had the same peak, and the peak intensity of the C=N-C was relatively low, which can be attributed to the adhesion of the polydopamine on the surface of the CNNS, which reduced the N element content detected on the surface of the CNNS. The results mentioned above illustrate the successful attachment of the dopamine to the surface of the CNNS.

### 3.2. Fractured Surface Morphologies

**Figure 2:**
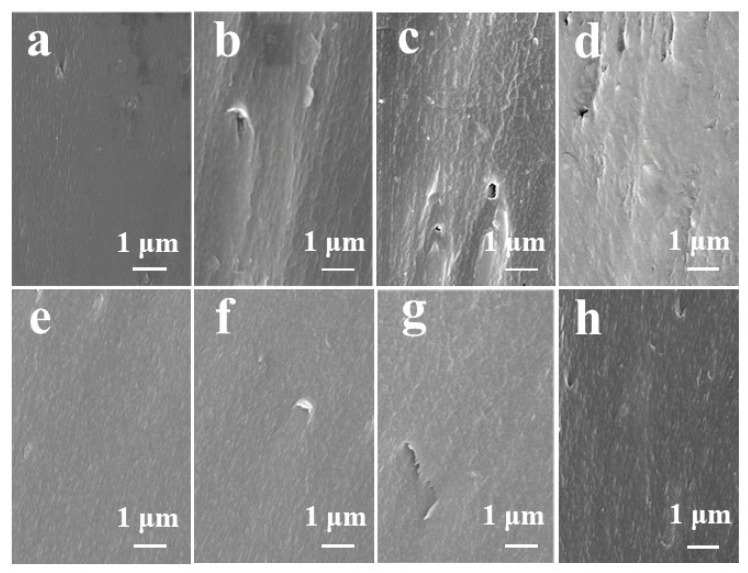
SEM images of the fractured surface for PI composites with (**a**) 0.25 wt%, (**b**) 0.5 wt%, (**c**) 0.75 wt% and (**d**) 1.0 wt% CNNS and (**e**) 0.25 wt%, (f) 0.5 wt%, (**g**) 0.75 wt% and (**h**) 1.0 wt% DCNNS.

The SEM images of the fracture surface for the PI composite films are shown in Figure 2. For the PI/CNNS composite films, some voids and defects can be seen in the composites, indicating the poor interfacial compatibility between the CNNS and the PI that becomes worse with higher filler content. As for the PI/DCNNS composites, no voids or defects can be seen in the composites with 0.25 wt% and 0.5 wt% filler contents. Moreover, some voids and defects were observed in the composites with 0.75 wt% and 1.0 wt%, but still much fewer than those in the PI/CNNS composites with the same filler content. Only when the specific gravity of the DCNNS reached 1.0 wt% did a small number of protrusions appear, while the PI film with the CNNS added did cause filler agglomeration at 0.75 wt%. This can be attributed to the fact that the hydroxyl groups on the surface of the DCNNS can form hydrogen bonds with the PI matrix or covalent bonds with the carboxyl groups of polyamic acid (the medium product used during the synthesis of the PI). This provides a morphological basis for the better breakdown performance of the PI/DCNNS composites, as discussed below. Under the action of the high-voltage electric field, such structural defects in the composites might induce the distortion of the electric field, which would directly affect the breakdown characteristics of the composite materials.

### 3.3. Dielectric Performance

Breakdown strength is an important parameter used to evaluate dielectric materials.

Due to differences in structure and process, the scatter of breakdown times and breakdown strength may be large even for identical specimens. The researchers found that the breakdown voltage obeyed statistical probability distribution under the test standard [31]. The Weibull distribution is a statistical distribution proposed by the Swedish physicist Weibull in the fatigue test of materials, the main feature of which is to find the weakest independent small unit in the material and to calculate it analytically [32]. It is generally believed that the breakdown of polymer-insulating material occurs at the weakest point of the polymer. Therefore, in dielectric physics, the Weibull distribution is commonly used for statistical analysis of the breakdown field strength of polymer-insulating materials under DC and AC voltages [33]. The statistical model of the Weibull distribution can be used to analyze the magnitude and dispersion of the breakdown field strength of dielectric materials and to obtain the stability analysis results of the breakdown of dielectric materials.

The breakdown strength of the CNNS and DCNNS composites were analyzed by two parameter Weibull distribution approaches, which reflected the probability of the material being broken down under a certain electric field and the probability of failure after a certain electric field action time, as depicted in this formula:

$$P\left(E\right)=1-exp\left[-{\left(\frac{E}{E_{b}}\right)}^{\beta }\right]$$

where P(E) is the cumulative probability of failure for electrical faults, E is the breakdown field strength for each data point, $E_{b}$ is the characteristic breakdown field strength where the cumulative failure probability is 63% for the polymer, and β is the Weibull shape parameter used to assess the dispersion of the experimental data. In this experiment, for each sample, 11 data points were taken for testing to estimate the corresponding Weibull breakdown strengths.

**Figure 3:**
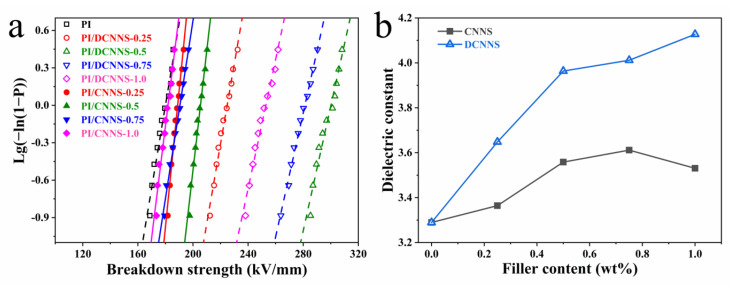
(**a**) Weibull distribution of electric strength and (**b**) dielectric constant change law of PI composites.

Figure 3a shows the Weibull distribution DC breakdown strength of the pure PI and PI composite films. As shown in Figure 3a, the breakdown strength of all the PI/CNNS composite films was a little higher than that of the pure PI (~179 kV mm^−1^). Nevertheless, all the PI/DCNNS composite films exhibited much higher breakdown strength than the PI/CNNS composite films with the same filler content, certainly significantly higher than the pure PI. In addition, as the content of DCNNS increased, the breakdown strength of the PI/DCNNS composite films first increased and then decreased. The PI/DCNNS composite film containing 0.5 wt% DCNNS showed the highest breakdown strength of ~300 kV/mm, which is 67.6% and 14.5% higher than the pure PI and the PI/CNNS with the same filler content, respectively.

Owing to the high specific surface area of the two-dimensional nanomaterials, the interface between two-dimensional nanofillers and polymers occupies an important position even at low-filler content [34]. The interface layer surrounding the nanofiller is believed to dissipate the charge and improve the internal electric field distribution, while the dispersed nanofiller acts as a scattering center to help reduce the charge transport. The deep interface traps, introduced by incorporating the nanosheets, can capture carriers, leading to a decrease in mobility and thus an increase in the electrical strength at the working frequency. Compared with the PI/CNNS composite films, the stronger interfacial interaction results in much fewer defects in the PI/DCNNS composite films, and the tight combination of the PI and nanosheets also reduced the fluidity of the PI macromolecular chain and the carrier transport. Furthermore, more polar groups on the surface of the DCNNS enhanced the electron scattering. Therefore, a higher breakdown strength for the PI/DCNNS composite films was observed. The decrease in breakdown strength for filler content above 0.5 wt% should be due to the formation of the filler agglomerations in the PI matrix.

Moreover, the dielectric constant of PI composite films with various filler contents at 1 kHz is shown in Figure 3b. All the PI/DCNNS composite films exhibited a higher dielectric constant than the PI/CNNS composite films with the same filler content, which should also be attributed to the stronger filler–matrix interactions and better filler dispersion.

## 4. Conclusions

In this work, the DCNNS were prepared using ultrasonic stripping of the bulk g-C_3_N_4_ followed by polydopamine modification, which was confirmed by XPS. PI composite films with various contents of CNNS and DCNNS were prepared via the in-situ polymerization method. The modification of the g-C_3_N_4_ by polydopamine was revealed to strengthen the interfacial compatibility between the PI matrix and the DCNNS, and reduce the defects in the composites, resulting in an improvement in breakdown strength. Compared with the pure PI film, the PI/DCNNS composite films showed increased dielectric constant and a much higher breakdown strength. The PI/DCNNS composite film with 0.5 wt% DCNNS showed an increase in breakdown strength by 67.6% and 14.5% as compared to the pure PI and the PI/CNNS composite film with the same filler content, respectively. Therefore, we provided a simple and convenient method to prepare high-voltage insulating materials with excellent breakdown strength and good dielectric properties by adding only a small amount of nanosheets.

## Data Availability

Not applicable.
